# The role of feedback in amblyopia treatment – a multi-centre randomised control trial

**DOI:** 10.1038/s41433-026-04383-7

**Published:** 2026-03-12

**Authors:** Gail D. E. Maconachie, Michael Hisaund, Seema Teli, Ashleigh Mellors, Lucy Mallory, Mansha Seewoodharry, Viral Sheth, Ravi Purohit, Rebecca J. McLean, Julie Kempton, Shegufta Farooq, Alison Bruce, Frank A. Proudlock, Irene Gottlob

**Affiliations:** 1 The University of Leicester Ulverscroft Eye Unit, Department of Psychology and Visual Sciences, University of Leicester, RKCSB, PO Box 65, Leicester, LE2 7LX UK; 2 School of Allied Health Professionals, Nursing and Midwifery, Faculty of Health, University of Sheffield, Sheffield, UK; 3 Clinical Engineering, University Hospitals of Leicester, Leicester, UK; 4 Bradford Teaching Hospitals, Bradford, UK; 5 Attending Physician, Neuro- and Pediatric Ophthalmologist, Department of Neurology Cooper University Health Care, Professor of Neurology, Cooper Medical School of Rowen University, Camden, NJ USA

**Keywords:** Outcomes research, Paediatrics

## Abstract

**Background:**

Conventional occlusion is an effective treatment for amblyopia; however, adherence remains a significant barrier. This is the first randomised controlled trial (RCT) investigating whether objective, electronically monitored adherence “feedback” improves amblyopia treatment adherence.

**Methods:**

This unmasked, parallel two-armed multicentre RCT included 102 children aged 3-8 yrs with monocular amblyopia (≥0.3 logMAR interocular difference). Participants could have up to 18 months of previous patching and were prescribed 10 h/6days of patching. Participants were randomised to a Feedback Group (*n *= 51), receiving feedback from treatment monitors, or Controls (*n* = 51). Change in adherence (CA) was measured from the first to last available monitor for patching and glasses over 12 weeks. Regression models explored factors influencing visual outcome and adherence.

**Results:**

Of 102 participants, 74 were analysed for patching and 78 for glasses. Mean patching CA was -0.39 ± 2.01 h/day (control) versus -0.32 ± 2.20 h/day (feedback), with no significant group difference (*P* = 0.89). Median glasses-wearing CA was -0.55 IQR:2.55 h/day (control) vs. -0.05 IQR:1.73 h/day (feedback), also non-significant (*P* = 0.38). Overall average adherence to glasses was 10.3 h/day and 7.9 h/day for patching. Younger age, less previous patching, and higher adherence to treatment significantly predicted better visual outcome. Females had significantly lower glasses-wearing adherence.

**Conclusion:**

This study shows for the first time that patching and glasses adherence can be monitored and fed back to patients and their carers. While we found no additional influence of feedback on adherence, we observed that when children and their guardians were aware of active monitoring and frequently seen, we observed high and sustained levels of adherence. The significant correlations to visual outcomes further highlight the importance of early treatment in amblyopia.

## Introduction

Amblyopia, with an estimated prevalence of 2.5–3.6% [1, 2], accounts for a significant proportion of paediatric eye clinic visits. Untreated, it leads not only to a significant deficit in visual acuity (VA), but also to other potential negative consequences, including increased lifetime risk of loss of vision in the non-amblyopic eye [3], diminished reading proficiency [4] and impairments in both fine and gross motor skills [5].

Despite interventions such as interactive computer-based dichoptic stimulation [6] and pharmacological intervention [7], conventional treatment, involving glasses wear (optical treatment) and occlusion, remains predominant worldwide. The most effective treatment period is during the time-limited plasticity of the developing visual cortex in childhood. Research by various groups, including the Paediatric Eye Disease Investigator Group (PEDIG), and Proudlock et al. [8] has confirmed the efficacy of conventional amblyopia therapy. However, adherence to treatment remains a barrier to effective visual improvement. As shown by objective dose monitors, participants achieve approximately half of the prescribed number of hours per day [9–12]. Sub-optimal adherence prolongs treatment and leads to poorer visual outcomes, increasing the burden on patients, guardians and healthcare systems.

Objectively monitored patient treatment adherence with the data from monitors being given back to patients, often termed “feedback”, has been trialled successfully in other diseases such as asthma or diabetes, leading to improved and sustained adherence over time [13]. While previous work [10, 12, 14, 15] has demonstrated the feasibility of electronic monitors to measure the duration of patching and glasses wear objectively [16]. This data has not yet been used for participant feedback.

This is the first randomised controlled trial (RCT) to investigate whether providing objective adherence data (“feedback”) from electronic monitors can improve adherence to patching and glasses wear. The primary aim was to determine whether feedback improves patching adherence. Additionally, we explored whether feedback improves glasses-wearing adherence, and finally, we examined whether any demographic or adherence-related factors could predict visual outcomes and treatment adherence.

## Material and methods

This multicentre trial was conducted at three UK NHS Trusts (University Hospitals of Leicester, Leicestershire Partnership, and Bradford Teaching Hospitals) from 2013 to 2019. Ethics committee approval was obtained, and the study adhered to the Declaration of Helsinki. The trial is registered at www.isrctn.com (ISRCTN85110914). Written informed consent was obtained from each participant’s parent or guardian.

### Patient selection

Eligibility criteria were children aged 3.0 to <9.0 years with monocular amblyopia (anisometropia, strabismus or mixed amblyopia); a minimum interocular VA difference of 0.3 logMAR, with VA in the amblyopic eye worse than 0.3 logMAR, and a minimum VA in the sound eye of 0.3 logMAR. Participants could have undergone previous patching for up to 18 months. Exclusion criteria were bilateral amblyopia, prematurity (<36 weeks gestation), and other ophthalmic or neurological disease. All participants had a full ophthalmological and orthoptic examination and cycloplegic refraction prior to inclusion into the study.

### Study design and randomisation

**Fig. 1:**
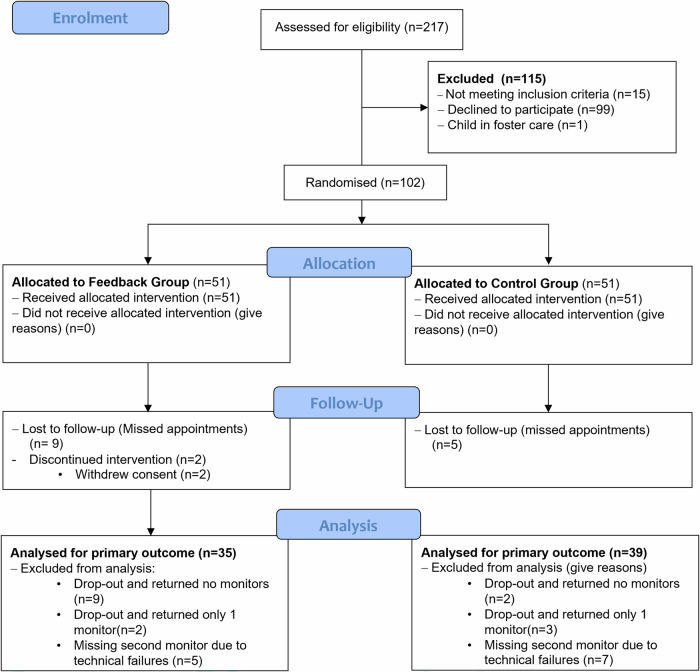
CONSORT flow diagram of participant progression through study phases. This flowchart illustrates the movement of participants through the four primary stages of the trial: enrolment, allocation, follow-up, and analysis. Throughout the diagram, the symbol n denotes the number of participants.

**Fig. 2:**
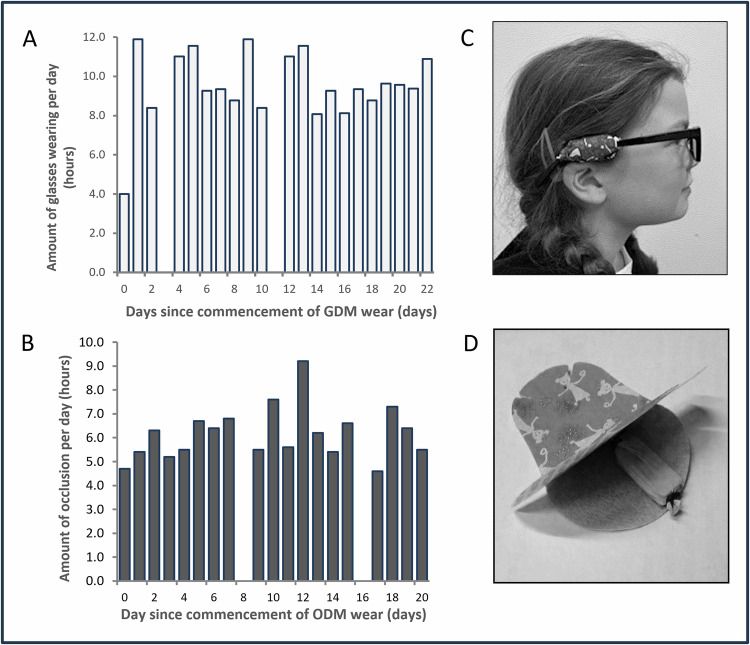
Implementation of glasses dose monitors (GDM) and occlusion dose monitors (ODM) for treatment adherence monitoring and clinical feedback. **A** displays representative daily glasses-wearing duration (h) over a 22-day period recorded by the GDM for a participant demonstrating fair adherence. **B** shows representative daily occlusion (patching) duration (h) over a 20-day period recorded by the ODM for a participant demonstrating good adherence. **C** illustrates the GDM device mounted on the temple of a child's spectacles. **D** depicts a clinical eye patch with the ODM device secured internally. Data derived from these monitors were presented to parents and children to facilitate informed discussions and encourage treatment compliance, particularly in instances where recorded adherence was lower than prescribed (i.e., (**A**) day 3 and day 11).

This unmasked parallel two-armed RCT (Fig. 1) enrolled 102 participants, randomised equally into a feedback group and a control group. The feedback group received objective adherence information from Electronic Dose Monitors (EDMs), presented graphically as hours per day (Fig. 2A and 2B). Randomisation was stratified by duration of previous occlusion (0-3, 4-6, 7-12, 12-18 months), age, and amblyopia severity (mild-moderate: VA in amblyopic eye better than 0.600 logMAR and severe as 0.600 logMAR or worse). Randomisation was performed via an Excel list by a remote, masked randomiser (RJM).

### Treatment protocol

All participants followed the same treatment protocol: glasses were prescribed to be worn during all waking hours, and the non-amblyopic eye was patched for 10 hours per day, 6 days per week. This follows previously reported intense and effective patching protocols by our group [8, 12, 16]. Patching hours were reduced if the amblyopic eye was successfully treated (≤0.1 logMAR interocular VA difference) or if adverse side effects (e.g., diplopia, increased strabismus) occurred. Nine participants had reduced patching. For analysis, patching hours were recalculated as a proportion of prescribed hours, scaled to a value relative to 10.

### Electronic dose monitors

Glasses and patching adherence were electronically monitored using the EDMs: the glasses dose monitor (GDM) for glasses wearing, and the occlusion dose monitor (ODM) for patching. A further description of the EDMs is outlined in previous studies [8, 16]. EDMs were developed with the Clinical Engineering department at the University Hospital of Leicester. EDMs obtained measurements by detecting the temperature on each surface of the monitor. Temperature readings were taken every 10 min, with a temperature resolution of 0.125°C. Data was analysed using both absolute temperature data and temperature differences between surfaces to differentiate wear from non-wear periods using operator-defined linear equations. Readings were downloaded via proprietary software and analysed in Microsoft Excel (2010) to calculate daily wear hours and generate graphical charts for the feedback group. GDMs were attached to glasses temples (Fig. 2C); ODMs were placed in a pocket formed by two occlusion patches (Ortopad Elite) (Fig. 2D). EDMs were replaced at each visit.

#### Examination procedures and follow-up

Participants underwent five orthoptic examinations over 12 weeks, at baseline (week 0) and then at three-weekly intervals (weeks 3, 6, 9, and 12; ±1 week).

To ensure that data could be reliably obtained and fed back to participants, three weekly examinations were prescribed. This was based on our previous studies and experience using these monitors [10, 16]. The examination was conducted by an orthoptist masked to which group the participant was allocated and included: uniocular VA (Crowded Keeler logMAR), cover test (near/distance, with/without correction), ocular motility, convergence, Bagolini glasses, and near stereoacuity (Frisby Near Stereotest). At each visit, EDMs were retrieved for data acquisition, and new monitors were provided. A second examiner downloaded and calculated adherence data while the orthoptic examination occurred. The feedback group received graphical EDM data (Fig. 2A and B) to inform discussion on adherence improvement. The feedback process followed a semi-standardised format and was delivered by the same experienced orthoptist at each visit. Feedback consisted of a brief explanation of the bar charts and average hours per day derived from each monitor, followed by a discussion of days with lower recorded wear time. Parents/guardians and children were invited to ask questions to clarify any concerns. Feedback was primarily directed at the parent/guardian, with the child included when considered developmentally able to understand the information; explanations were adapted to be age-appropriate where applicable. The content and duration of the discussions were consistent across participants, with each feedback session limited to a maximum of 10 min. The control group received standard adherence encouragement without EDM data. After 12 weeks, participants returned to routine clinical care.

#### Statistical analysis

The sample size was based on previous studies [10, 16] where the mean compliance was 66.19% ±SD 25.32% for patching and 64.29% ±SD 22.48% for glasses wear. To detect a difference of 15% (20%) in compliance between the two groups required 51 participants in each arm (α = 0.05, power = 80%, drop-out rate = 10%).

### Primary outcome measure

Change in patching adherence (average hours/day on days patched) over 12 weeks between groups. Change in Adherence (CA) from first to last available monitoring period was calculated for each individual (using monitors with the greatest time gap if planned intervals were missed due to technical faults or non-return). CA was compared using Mann-Whitney U test or independent t-test (based on data normality). Kruskal-Wallis test explored whether feedback session number (1-3) or interval (3-9 weeks) influenced adherence levels. Participants with only one or no valid monitoring periods were excluded from analysis.

Multiple linear regression explored which demographic factors (age, amblyopia type, previous patching, socioeconomic factors, severity, sex) affected CA (first to last monitor). Socioeconomic factors were assessed using location and the Index of Multiple Deprivation (IMD) [17], a UK government dataset combining 7 domains for deprivation ranking.

### Secondary outcome measures

Included analyses comprised of change in adherence to patching between the first and second monitoring periods, change in adherence to glasses wear between the first and last monitoring periods (and between the first and second monitoring periods), and predictive factors for visual outcome and adherence (glasses and patching). Glasses adherence change was analysed similarly to patching. Predictive models used step-wise multiple regression for visual outcome (percentage deficit corrected, per Stewart et al. [15]) average patching adherence (hrs/day), and average glasses adherence (hrs/day). Factors included age, sex, amblyopia type/severity, and IMD. For visual outcome, glasses/patching adherence were also included. When predicting visual outcome, for participants with missing VA, VA was estimated using Last Observation Carried Forward (LOCF). Significant factors in regression were further explored via appropriate statistical tests after a normality check. Multiple comparisons were adjusted with Bonferroni correction.

Chi-squared test examined group differences in dropout rates. Significance for all statistical models was set at *P* < 0.05.

## Results

### Participants

One hundred and two patients (51 feedback, 51 control) were recruited (Fig. 1). Baseline characteristics are in Table 1. Initial amblyopic eye distance VA was not significantly different between feedback (0.663 ± 0.233) and control (0.698 ± 0.267) groups (*P* = 0.50). Overall, 16 children dropped out of the study (11 feedback, 5 control), the difference between groups was not significant (X^2^(1, 102) = 2.67; *P* = 0.10). Reasons for dropout in the feedback group included no reason (7), monitor-related (3), and psychosocial issues (1). In the control group, 4 gave no reason, and 1 struggled with patching. Eighty-six participants completed the trial.

**Table 1:** Demographics of all participants in the feedback and control group, with absolute numbers and percentages in brackets unless otherwise stated.

	Feedback (*n* = 51)	Control (*n* = 51)
**Age [years] (SD)**	5.5 (1.6)	5.6 (1.4)
**Sex: Female**	24 (47.1%)	21 (41.2%)
**Ethnicity**		
White British	44 (86.3%)	36 (70.6%)
White Irish	3 (5.9%)	1 (2.0%)
White Other	1 (2.0%)	3 (5.9%)
Asian	3 (5.9%)	6 (11.8%)
Chinese	0 (0.0%)	0 (0.0%)
Black British	0 (0.0%)	2 (3.9%)
Mixed (White British/Black British)	0 (0.0%)	1 (2.0%)
Mixed (White British/Asian)	0 (0.0%)	2 (3.9%)
**Refractive Adaptation (weeks)**	49.6 (Ra:0-233)	48.3 (Ra:1-268)
**Prior occlusion treatment**	16 (31.4%)	22 (43.1%)
**Duration of Previous Occlusion (Months)**		
0- < 3	42 (82.4%)	43 (84.3%)
3- < 6	5 (9.8%)	2 (3.9%)
6- < 12	3 (5.9%)	4 (7.8%)
12-18	1 (2.0%)	2 (3.9%)
**VA in Amblyopic Eye [mean logMAR](SD)**	0.680 (0.258)	0.697 (0.264)
**VA in Fellow Eye [mean logMAR](SD)**	0.077 (0.102)	0.090 (0.100)
**Interocular difference [mean logMAR](SD)**	0.602 (0.234)	0.607 (0.247)
**Amblyopia Severity**		
Moderate	20 (39.2%)	18 (35.3%)
Severe	31 (60.8%)	33 (64.7%)
**Amblyopia Aetiology**		
Strabismic	11 (21.6%)	15 (29.4%)
Anisometropic	19 (37.3%)	16 (31.4%)
Mixed (Anisometropic and Strabismic)	21 (41.2%)	20 (39.2%)

### Deviations from protocol

All 86 participants were prescribed 10 h of occlusion per day, 6 days per week. Two control group participants had patching briefly paused due to apparent inverse amblyopia, which was later attributed to limited cooperation with VA assessment; occlusion restarted as per protocol.

### Monitors

A total of 369 ***ODMs*** were given to the 102 participants. In the feedback group, 178 ODMs were given, of those that were returned and used (150 ODMs; 26 not returned, and 2 not used), 31 failed, giving a success rate of 79.3% (119/150). In the control group, 191 ODMs were given; of those that were returned and used (167 ODMs; 23 were not returned and 1 was not used), 23 failed giving a success rate of 83.8% (140/167). The total success rate for ODMs was 81.7%. A total of 374 ***GDMs*** were given to the participants. In the feedback group, 180 monitors were given; of those which were returned (134 GDMs; 16 were not returned), 30 failed, giving a success rate of 81.7% (134/164). For controls, 194 monitors were given with 32 failing, giving a success rate of 82.2% (148/180; 14 were not returned). The total success rate for GDMs of 82.0%.

### Change in adherence to patching

**Fig. 3:**
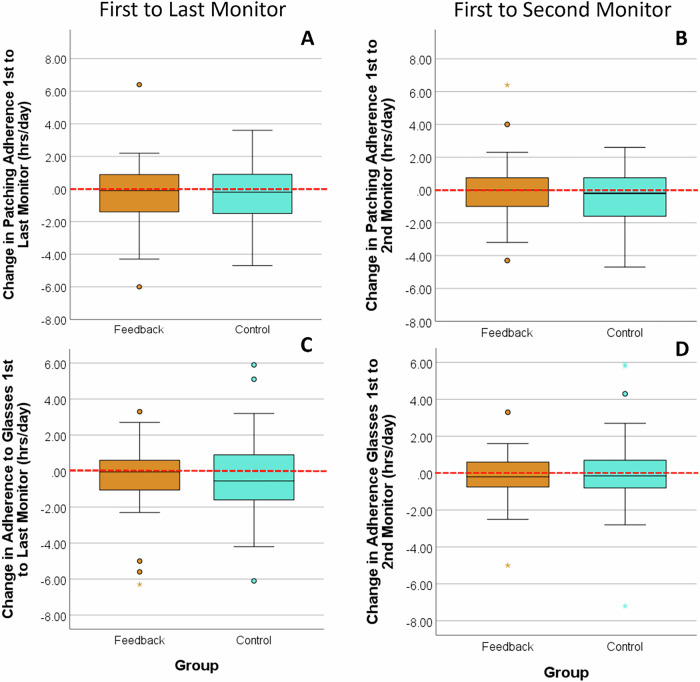
Box Plots of Adherence Changes. Box plots illustrate changes in patching (3 A – 1^st^ to last patch monitor, 3B – 1^st^ to 2nd patch monitor) and glasses adherence (3 C – 1^st^ to last glasses monitor, 3D- 1^st^ to 2nd glasses monitor) between the control (turquoise) and feedback (orange) groups. The box represents the interquartile range (IQR), with the median shown as a horizontal line. Whiskers extend to values within 1.5 times the IQR, while circles (○) and asterisks (*) denote mild and extreme outliers, respectively. The red dashed line marks no change in adherence.

The primary outcome analysis included 74 participants (39 control, 35 feedback) with at least two ODM readings. In the feedback group, 12 participants received three sessions of feedback, 14 two sessions, and 9 one. There was no correlation between the number of feedback sessions and adherence levels (*P* = 0.45) or time between 1^st^ and last monitor (*P* = 0.84). Mean patching CA from first to last monitor was -0.39 ± 2.01 h/day (control) and -0.32 ± 2.20 h/day (feedback). There was no significant difference in patching CA between groups (t(72) = -0.133; *P* = 0.89; Fig. 3A), even after adjustment for confounders. The overall decrease in adherence (first to last monitor) across all participants was insignificant (*n* = 74,Z = − 1.226; *P* = 0.22).

Mean CA between first and second monitor was -0.52 ± 1.74 h/day (control) and +0.03 ± 1.91 h/day (feedback), with no significant group difference (t(72)= -1.290; *P* = 0.20, Fig. 3B) and remained insignificant after adjustment.

### Change in adherence to glasses wearing

Seventy-eight participants (42 control, 36 feedback) had at least two GDM readings. In the feedback group, 20 participants received 3 sessions of feedback, 12 two and 4 one. The number of feedback sessions did not correlate with adherence levels (P = 0.39) or time between feedback (*P* = 0.70). Median CA between first and second monitor was -0.15 IQR: 1.53 h/day (control) and -0.20 IQR: 1.38 h/day (feedback). Between first and last monitor, median CA was -0.55 IQR: 2.55 h/day (control) and -0.05 IQR: 1.73 h/day (feedback). No significant difference was found between groups for CA (first to last: U = 669, Z = -0.872; *P* = 0.38; Fig. 3C or first to second: U = 713, Z = -0.421; *P* = 0.67; Fig. 3D), even after adjustment. Overall change from first to last glasses monitor was insignificant (*n* = 78, Z = − 1.686; *P* = 0.09).

### Visual outcome and dose-response

Average previous glasses wear before entry into study was 44.3 weeks in the feedback group and 47 weeks in the controls. Visual improvement after 12 weeks of patching was 3.59 logMAR lines (SD ± 2.01 lines) in the feedback group and 3.14 logMAR lines (SD ± 1.93 lines) in controls (difference: *P* = 0.77). The corrected deficit was 58.9% in the feedback group and 54.3% in controls (difference: *P* = 0.48). In participants with no prior patching treatment, this increased to 67.5% and 66.9%, respectively. Moderately amblyopic participants (<0.600 logMAR amblyopic eye) without prior treatment improved by 2.96 logMAR lines (SD ± 1.29), while severely amblyopic (>0.600 logMAR amblyopic eye) participants improved by 4.64 logMAR lines (SD ± 1.91).

**Fig. 4:**
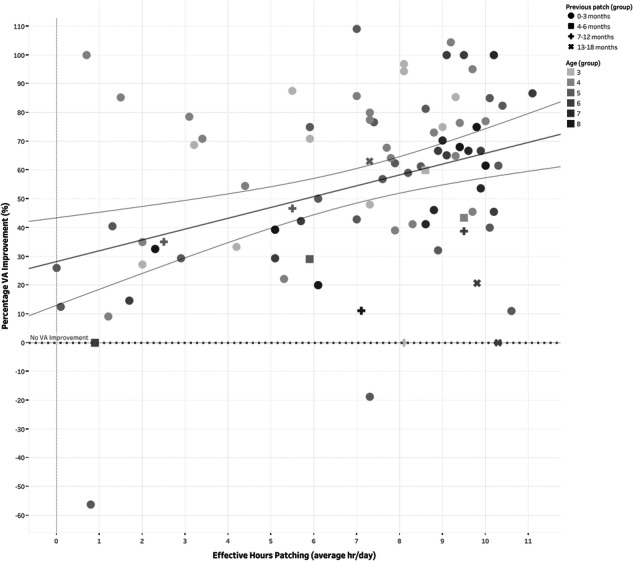
Scatterplot showing the relationship between effective hours patched (hrs/day) and percentage improvement in visual acuity (visual outcome). Individual points are grouped by age (progressive darkening of blue colour where 3 yrs old is the lightest and 8 yrs old the darkest) and prior patching treatment in months, indicated by shape (circle – 0-3months, square – 4-6months, plus – 7-12months and x – 13-18months). The solid trend line represents the overall relationship, with the area between the outer grey lines showing the 95% confidence intervals of the mean. The red dotted line indicates % improvement in VA.

A stepwise multiple linear regression model (*n* = 85) predicted visual outcome (R^2^ = 0.341, F(4, 81) = 10.47; *P* < 0.001). The model was: y = 36.944 − (3.03×previous patching in months) + (2.62×adherence to patching) - (5.33×age) + (3.21×adherence to glasses wear). Lower previous patching, younger age, and higher glasses and patching adherence significantly predicted better VA outcome (Fig. 4). Sex, IMD, initial amblyopic eye VA, and amblyopia type did not influence VA outcome.

### Predictors for treatment adherence

A significant positive moderate correlation existed between patching and glasses adherence (*r* = 0.549; *P* < 0.001, *n* = 86 – Supplementary file 1). Glasses adherence (median=10.3 h/day) was higher than patching adherence (median=7.9 h/day). A stepwise regression model for patching adherence was insignificant, with no factors contributing. For glasses adherence, the fitted model was y = 10.326 + ( − 1.28×Gender), and was significant (R^2^ = 0.065, F(1, 89) = 6.23; P = 0.014). Females had significantly lower glasses adherence (9.63 h vs. 10.89 h for males; U = 646, Z = -2.951; *P* = 0.003, Supplementary file 2).

### Adverse events

No serious adverse events occurred. Two control group participants had a temporary vision drop in the non-amblyopic eye attributed to cooperation issues, not treatment.

## Discussion

This study marks the first multicentre RCT using EDMs to provide feedback on patching and glasses wearing adherence during treatment to participants and their carers, with a success rate of over 80%. While previous research has provided valuable insight that patching adherence is variable (average 50% of prescribed hours) [12] and that intervention materials can improve it [10, 18]. Our study found that, although there was evidence that monitoring adherence produces high and sustained levels of adherence through knowledge of active monitoring, there was no significant effect of feedback from EDM monitors. This finding is contrary to findings in other specialities (e.g. asthma, medication [13]) who have successfully used objective monitoring feedback to improve adherence, including in children [19, 20].

Several factors may explain the lack of additional benefit from feedback. Importantly, both groups achieved high levels of adherence to patching, exceeding 70% of prescribed levels. This adherence is substantially better than the typically reported in ODM studies (41.2% to 57.5%) [9, 12] and is comparable to groups that received additional educational intervention materials (60.3% to 84.5%) [10, 18]_._ This overall high adherence, which remained stable over the 12 weeks, suggests a ceiling effect for the feedback intervention, indicating that a substantial proportion of the prescribed dose was already being achieved. Nevertheless, given the minimal costs and ease of providing feedback, it may still be reasonable for clinicians to incorporate feedback into routine clinical reviews at their discretion, particularly for individuals at risk of poorer adherence. This high adherence was likely driven by the frequent 3-weekly clinical visits, which is substantially greater than the recently reported international average of 8.51 weeks (SD ± 4.58) [21], and the participants’ fundamental awareness of being actively monitored. Our research, therefore, highlights that an important focus for future research should be to explore further whether frequent clinical visits alongside objective monitoring are effective strategies for achieving and maintaining a high level of treatment adherence.

In our study, we observed a higher overall dropout rate than planned. The trend for a higher dropout rate in the feedback group (11 vs. 5), although not statistically significant, is an important finding that may reflect the difficulty poorly adherent patients and guardians have with intense monitoring and the pressure of feedback. This likely resulted in a self-selection of a highly adherent cohort remaining in the final analysis. Furthermore, due to the monitor failure and non-return, the final analysis of adherence was limited to 74 participants for patching and 78 for glasses. This limited sample size, alongside the pre-existing high adherence levels, likely results in an underpowered study that is statistically insufficient to detect small differences between groups. Accounting for a higher dropout rate and monitoring limitations in the sample size calculations for future research is essential.

Visual outcomes were also explored as part of this study, with the majority achieving nearly 3 lines of improvement in 12 weeks with intensive patching. In participants with no previous treatment, nearly 3 lines of improvement were achieved in children with moderate amblyopia and over 4.5 lines in the severe group. This appears more rapid than the improvements reported in the PEDIG study (2003) [22, 23], which found a 2.40-line improvement in their moderate group (prescribed 2 or 6 h of patching) and a 4.8-line improvement in their severe group (prescribed 6 or 12 h of patching) after 4 months. Direct comparison is limited due to differences in study protocols, endpoints (4 months vs. 12 weeks), and unmonitored adherence in PEDIG. Nonetheless, our findings suggest that, when adhered to, intensive treatment may accelerate visual improvement. Further research, however, is required to assess this in more detail, including the potential impacts on quality of life.

An important finding of our study is that more effective hours achieved in patching result in significantly better visual outcomes. In addition, this study also found that younger age and no previous treatment predicted better visual outcomes. As previous studies revealed that adherence to treatment declines with time [24], we hypothesised that previous patching would reduce adherence. However, we did not find that previous patching resulted in reduced patching or glasses adherence. Our finding that younger age resulted in better visual outcomes agrees with Holmes and Levi [25] and our previous study [8], which found that age is a consistent limiting factor in amblyopia treatment. These findings highlight the importance of detecting amblyopia early and achieving high levels of adherence at the start of treatment to achieve good outcomes.

We investigated potential risk factors that might lead to lower adherence. The only factor which we found to influence the amount of glasses wear was biological sex, with a slightly longer median time of wearing glasses in boys. There could be many potential reasons why females may be less adherent to glasses wear. In one study undertaken in India, non-adherence to glasses was associated with greater parental disapproval of glasses wear in females than males [26]. Further research would be important to detect the causes of poor adherence to glasses.

There are some potential limitations to this study. Firstly, as participants were seen every 3 weeks to ensure reliable data collection from monitors, this is less reflective of a clinical setting. Developing robust monitors that last reliably for 6-weekly or 2-monthly frequency may be more appropriate. The graphical presentation provided, while objective, might not have been optimally engaging for children or their carers. Future studies should explore child-friendly, digitally-enabled feedback, including more imaginative or cartoon-based graphics, gamification, or rewards to encourage adherence, similar to those seen with dichoptic treatment monitoring [27]. In other fields, such as paediatric asthma management, studies have shown that the addition of electronic reminders and remote real-time contact delivered by the patient’s treating clinician can significantly improve adherence and could be considered in future studies [28, 29].

This study has successfully shown that objective monitoring and feedback of glasses and patching adherence is feasible in a multi-centre setting, with and an overall monitor success rate exceeding 80% for both patching and glasses. While adherence feedback did not offer an additional benefit in this highly-adherent cohort, the study highlights several important clinical findings from our secondary outcome measures. Firstly, patching adherence remains critically important for good visual outcomes, as the effective amount patched per day was positively correlated with visual improvements. Secondly, the predictive factors of younger age and no previous treatment underscore the importance of early detection and treatment. Lastly, our findings that females had significantly lower glasses-wearing adherence identify a key demographic at risk for poorer adherence that requires further study and targeted intervention. Considering the sustained high levels of adherence achieved in both groups, this study suggests that frequent clinical visits alongside objective adherence monitoring are key components that effectively contribute to good adherence and, subsequently, better visual outcomes.

## Summary

### What was known before

- Adherence to amblyopia treatment remains a significant barrier to achieving good visual outcomes. While some previous studies have demonstrated that educational materials and interventions can improve adherence, this benefit has not been consistently or universally observed across all patient populations or settings. Objective feedback derived from electronic monitoring has shown success in improving patient adherence in other medical fields, but its use for improving adherence in amblyopia treatment remains unexplored.

### What this study adds

- This is the first trial to show that objective monitors can be effectively used to provide feedback on adherence levels to patients with amblyopia and their guardians Objective and active monitoring of adherence, with or without specific feedback, leads to consistently high adherence rates (over 80%). The study uniquely found that females have significantly lower glasses-wearing adherence than males, identifying a crucial new at-risk demographic.

## Supplementary information

Supplementary File 1

Supplementary File 2.

## Data Availability

The datasets generated during and/or analysed during the current study are available from the corresponding author on reasonable request.
