# Correction: The role of feedback in amblyopia treatment – a multi-centre randomised control trial

**DOI:** 10.1038/s41433-026-04508-y

**Published:** 2026-07-21

**Authors:** Gail D. E. Maconachie, Michael Hisaund, Seema Teli, Ashleigh Mellors, Lucy Mallory, Mansha Seewoodharry, Viral Sheth, Ravi Purohit, Rebecca J. McLean, Julie Kempton, Shegufta Farooq, Alison Bruce, Frank A. Proudlock, Irene Gottlob

**Affiliations:** 1 The University of Leicester Ulverscroft Eye Unit, Department of Psychology and Visual Sciences, University of Leicester, RKCSB, PO Box 65, Leicester, LE2 7LX UK; 2 School of Allied Health Professionals, Nursing and Midwifery, Faculty of Health, University of Sheffield, Sheffield, UK; 3 Clinical Engineering, University Hospitals of Leicester, Leicester, UK; 4 Bradford Teaching Hospitals, Bradford, UK; 5 Attending Physician, Neuro- and Pediatric Ophthalmologist, Department of Neurology Cooper University Health Care, Professor of Neurology, Cooper Medical School of Rowen University, Camden, NJ USA

**Keywords:** Outcomes research, Paediatrics

Correction to: *Eye* https://doi.org/10.1038/s41433-026-04383-7, published online 12 March 2026

In this article the author name ‘Maconachie GDE’ is incorrectly displayed as ‘D E Maconachie G’ and needs to be corrected.

The original article has been corrected.

