# Supplementary figures and images for: The role of feedback in amblyopia treatment – a multi-centre randomised control trial
Source: Eye (Lond). 2026 Mar 12;40(8):1232–9. doi: 10.1038/s41433-026-04383-7 (PMC13194784; doi:10.1038/s41433-026-04383-7)

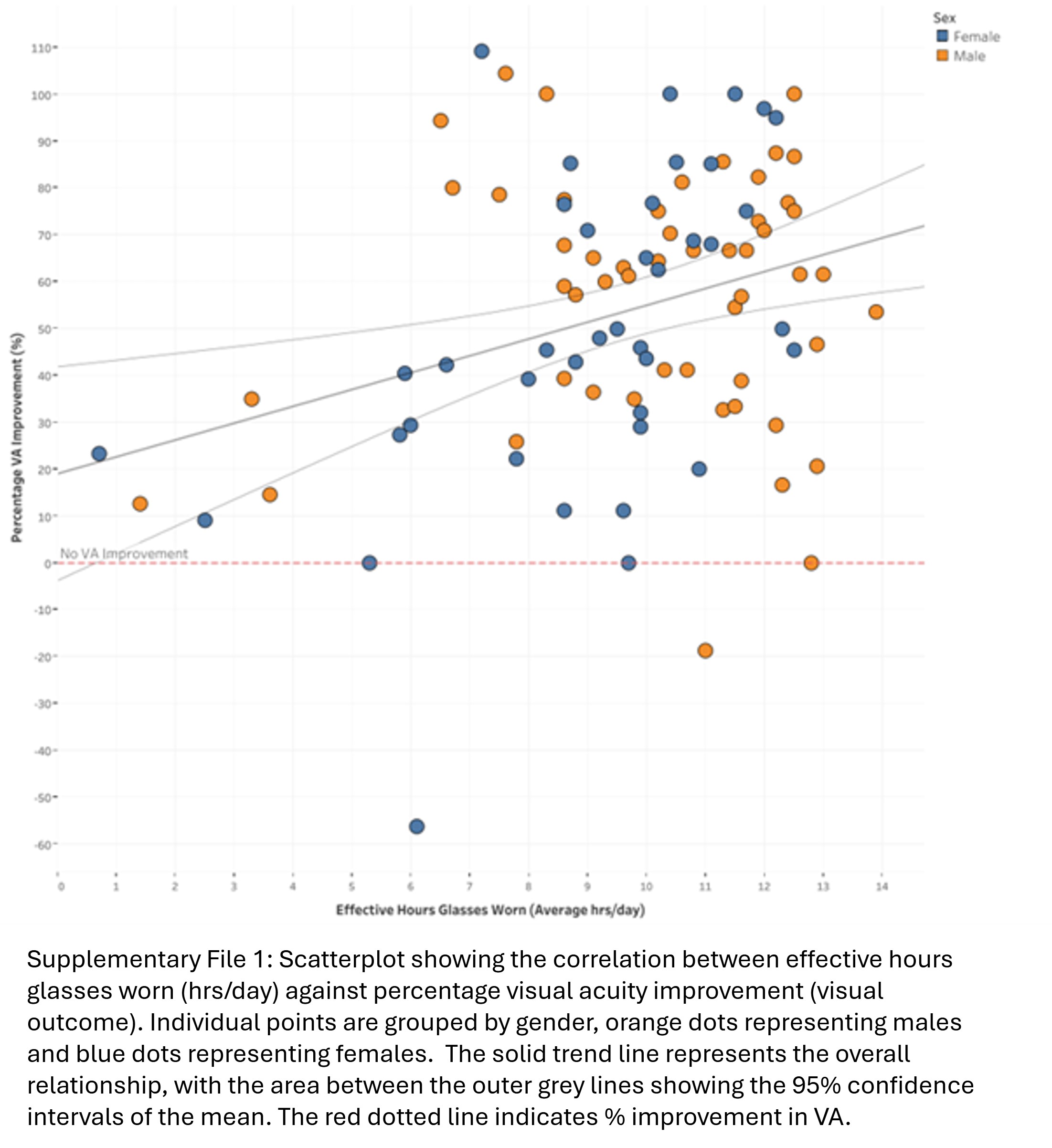

Supplement: Supplementary file 1 [file 41433_2026_4383_MOESM1_ESM.jpg]

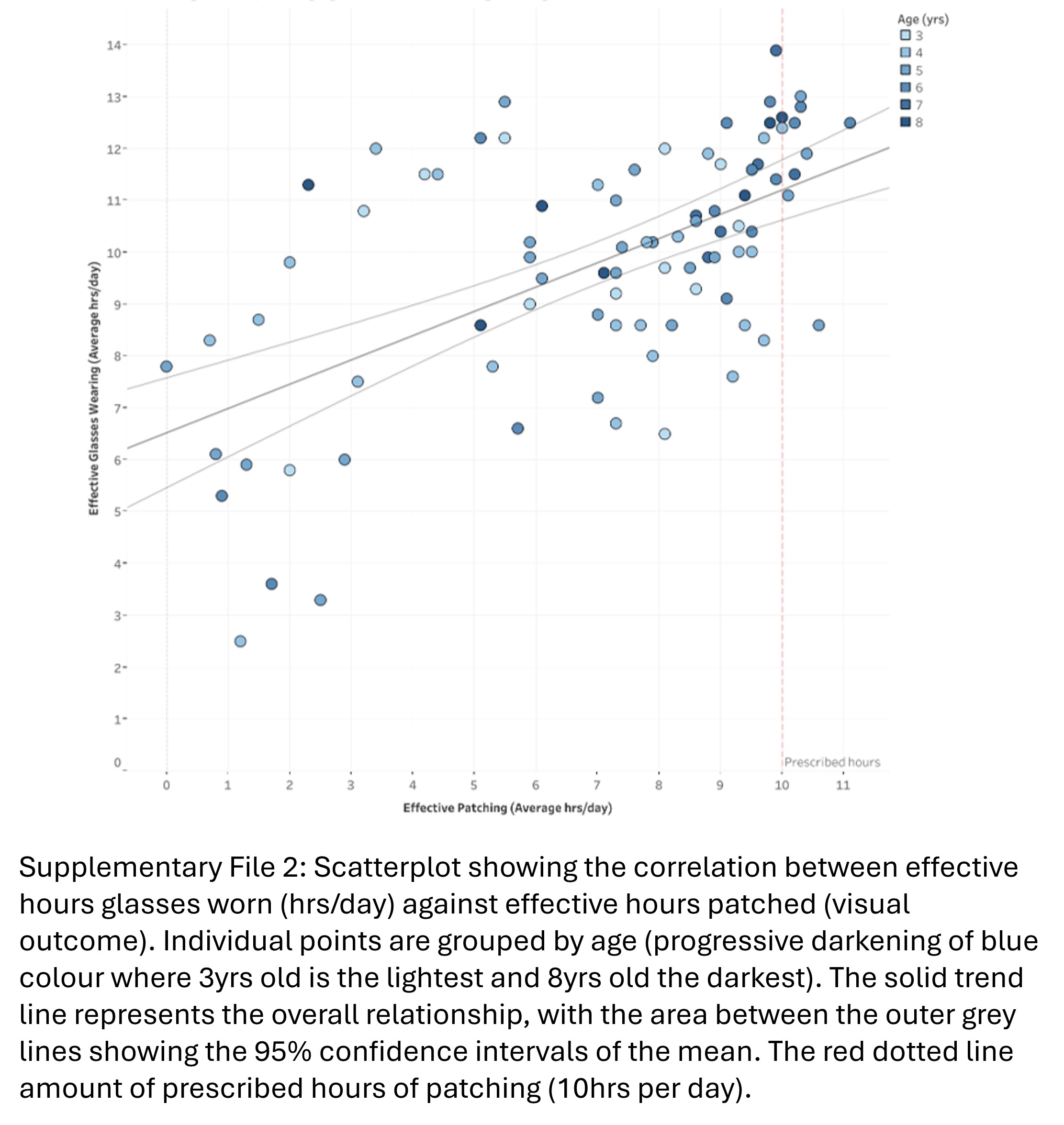

Supplement: Supplementary file 2 [file 41433_2026_4383_MOESM2_ESM.jpg]
